# Movement as a Means of Cure

**Published:** 1882-03-20

**Authors:** 


### MOVEMENT AS A MEANS OF CURE.

A natural outcome of the teachings of the expectant school of therapeutics, is the placing of rest in a high position among the means of cure. Granted that nature is able, unassisted by art, to restore the equilibrium between her parts which has been disturbed by the foreign element, disease, and it follows that all means designed to secure non-interference with the free play of nature's forces, are all essential. To deny that rest has no value in therapeusis would be practically equivalent to a denial of nature's power to heal, and the skeptics on this question have long since succumbed to nature's power to destroy. Nature is powerful but not omnipotent in the domain of therapeutics, and rest is a valuable but not an invaluable means of universal applicability. The dangers of hobbyism and extremism (empiricism) in the matter of application of rest have too many living illustrations, particularly in surgical practice, while very many more of its results are embowelled in mother earth. There is danger in all extremes, and the doctrine of rest, heralded though it has been by so many prophets and bards, has been no exception to this rule. Its danger has lain in its irrational application—the improper selection of cases, and its too prolonged continuance in cases in which it was at first indicated.

The antithesis of rest is movement, and as an offset to the pernicious results of rest the inculcation of the benefits of movement as a therapeutic agent would seem to be called for. The scientific lines between the necessity for the two are, it may be, difficult to trace, but the highest degree of success lies near those lines, and efforts to locate them should be unceasing. When has the inflamed joint or fractured limb been kept immobile just long enough, the inflamed peritoneum just long enough rested, the inflamed lung just long enough kept in an "opium splint?" These are questions on whose proper answer in individual cases successful therapusis largely hinges. In all cases which originally demanded rest there comes a time when rest must give way to motion. When is that time? is a question for the medical attendant to answer. Under enforced rest of the inflamed joint the effused synovial fluid has become absorbed and the symptoms of inflammation have disappeared; here is then the critical point. Shall the joint be longer kept at rest, as is the common practice, lest the possible dying embers be fanned into new life, or shall passive movement be practiced? Each individual case presents circumstances which must modify the decision as to such procedure, and it is in trimming sail to meet these circumstances that the skill of the medical attendant is displaced. That anchylosis on the one hand from too prolonged rest, and a renewal of inflammation from an insufficient continuation of such rest, are the Scilla and Charybdis in the treatment of joint affections, is attested by abundance of experience. The too cautious physician, by attempting to steer clear of one, allows the bark to be caught in the other, and it is to be feared that anchylosis as a result of too much rest is a too frequent result of treatment.

The necessity for movement, in the treatment of a diseased joint, having been determined on, the method of its performance becomes the practical question. In an article in the British Medical Journal, over a year ago, Dr. John Spender ably discussed this branch of the subject. He refers to a discussion of the subject, some years ago, by the late Mr. Hutton. Summed up in a few words, Mr. Hutton's principle was the rupture of adhesions by manipulative force, guided by skillful leverage. We learned from him that joints will bear much rougher usage than is sanctioned by professional usage.

In dealing with a joint which has not been materially damaged by inflammation, is not much enlarged, but is now weak mainly because the muscles are weak which ought to move it, a thorough and scientific shampooing (or massage) is our therapeutic watch-word. The physiology of the joint has to be studied more than its anatomy. The joint is afraid of itself because it is not used; its volitional energies are "hidden under a bushel;" and the owner of it has, perhaps, been scared by the terror of what may happen if he presumed to walk even with the help of crutches. It is satisfactory to know that Dr. Sayre, who is the designer of such an ingenious plan for supporting and resting a diseased spine, distinctly says that, in the case of anchylosed limbs, there is "a time for motion as well as a time for rest."

First, then, we satisfy ourselves that the joint-ends of two bones are not mortared together, and do not require any forcible methods for their separation. A patient may be put partially under the influence of chloroform if necessary, and the capability of a joint to be bent or extended will then be fairly tested, and we shall discover what checks there are to healthy movement. The passive play of a joint tells us that its mechanism is sound; but the question is: What are its vital activities? What can the patient do with it, and how far can he use it?

The simple plan of treatment which Dr. Spender has for years adopted is as follows: Imagine the knee to be the affected joint, and the foot should rest on a stool or block of wood just within a large shallow open bath, so that the knee is over nearly the centre of the bath. A jug, holding about a pint of fluid, is filled with tepid water, and is turned upside down about three feet above the knee, so that the water falls with a splash on the joint; and this is repeated from six to twelve times. An attendant, sitting in front of the patient, then plants a hand on each side of the knee; and, with the thumbs meeting in front, the hands should be moved firmly up and down for eight or ten minutes. The pressure used should be equal and well sustained, not causing any uneasiness, not in the least rough, but such an union of firmness and gentleness as a practical manipulator will easily understand. The thumbs, while agents of modern pressure themselves, may be made the fulcra for pressing and rubbing the back of the joint. At the end of the shampooing process, the whole joint ought to be dry and warm, and to be immediately wrapped in a covering of oiled silk lined with wadding, which should be securely fastened and held on for some hours.

By this easy plan, carried out regularly once or twice a day for several weeks, there is seldom any difficulty in restoring the torpid functions of non-anchlyosed joints. Now and then it may be desirable to suspend the friction for two or three days, if the skin shows signs of irritation; and in warm weather the impervious pad is scarcely necessary, and might cause an eruption of pustular acne. Medicated lotions or liniments are rarely prescribed; but now and then Dr. Spender introduces under the oiled skin wrap a piece of folded flannel soaked in a mixture of iodine (half an ounce), and soft water (seven ounces). The early douchings are best done with tepid water; but this should be exchanged for cold water as soon as possible, on account of the great glow and reaction which are afterwards obtained; and during the summer months, cold water may be used from the first. In all cases, the local treatment should be supplemented by regular passive movements carefully and coaxingly executed, and never exciting pain and fatigue. Sometimes it is only timidity which hinders a patient from (say) pronating and supinating a hand, or flexing and extending an elbow; a group of muscles have to be taught anew. As the lower limbs bear the weight of the body, their voluntary exercise must be deferred until the patient regains confidence and acquires strength.

The next stage of treatment consists in the application of uniform and gentle support. A weak joint requires support when it no longer requires rest. This seems to be the key of the new and important device of the American surgeons—prolonged pressure without prolonged rest. The principle is not at all new. And yet it ought to have been clear that to lay up a limb in idleness admits of the healing of an ulcer by a degree of vital action, which is unable to maintain the permanence of that healing when the limb is restored to use. Similarly, when we have to treat a weak joint, the principle ought to flash upon us with unerring instinct; here is a case, we should say, for applying firm pressure and allowing moderate exercise. For the ankle, there is nothing better than an India-rubber bandage, put on from the base of the toes to the upper part of the leg. The bandage is far better than a so-called elastic anklet, because it supports all the muscles which move the ankle, it should make a gentle uniform pressure, without any sensatiou of squeezing, each fold slightly overlaping the previous one all the way up the limb. The knee should be enclosed during the daytime in an elastic support, which should always be laced. The lacing allows the degree of support to be varied, according to the feelings of the patient and the amount of exercise he has to undergo. Equipped with this simple apparatus, the lower limb enjoys, as far as the upper part of the knee, a gentle and uniform pressure, which permits useful locommotion within restraining and salutary conditions.

The application of this principle to the upper limb is not difficult. A hand and wrist, when embraced by an elastic glove extending a little way up the arm, is competent to perform various feats of dextrous workmanship, which would otherwise soon cause aching and powerlessness. But what need is there for illustrating further such an obvious point of practice? If to be at work is the part which a healthy organ has to play, it is clear that too much rest cannot contribute to the perfection of that work, either in quantity or quality. That special condition which is called the "hysterical joint" is peculiarly amenable to the vigorous and helpful treatment of douching and pressure. For the douching, the use of the bath waters is much to be commended, as the dynamic stimulus of their heat maintains an after glow, which is a sign of more vital energy. And hysteria, if it mean anything, means a lack of this energy—a crippled volition, a sluggish blood stream, and a general under tone of life. The combination of firm pressure and moderate exercise fulfils, in this case, exactly what we ought to aim at: support to the mechanical apparatus of locommotion, and arousing and sustaining of vital function.

Rest and movement are, therefore, complementary to each other, both in physiology and in therapeusis. The analogy is as close as possible. Alternation of work and rest is the law of the human organism in health, and health could not be preserved without it. Disease may call for a prolongation of the element of rest; but it is a note of clinical insight to discover when the disease ends, or when sufficient health returns to justify the usual alternate rest and work. Continued work without rest could not be; rest continued too long is not only conceivable, but it is one of the present dangers to therapeutic surgery.—*Therapeutic Gazette.*

